# Role of the Unfolded Protein Response in ****β**** Cell Compensation and Failure during Diabetes

**DOI:** 10.1155/2014/795171

**Published:** 2014-04-09

**Authors:** Nabil Rabhi, Elisabet Salas, Philippe Froguel, Jean-Sébastien Annicotte

**Affiliations:** ^1^European Genomic Institute for Diabetes (EGID), CNRS UMR 8199, Lille 2 University of Health and Law, 59000 Lille, France; ^2^Departments of Genomics of Common Disease, Hammersmith Hospital, Imperial College London, London, UK; ^3^Laboratoire Bases Moléculaires et Modélisation du Diabète et de l'Obésité, Faculté de Médecine, Pôle Recherche, 59045 Lille, France

## Abstract

Pancreatic **β** cell failure leads to diabetes development. During disease progression, **β** cells adapt their secretory capacity to compensate the elevated glycaemia and the peripheral insulin resistance. This compensatory mechanism involves a fine-tuned regulation to modulate the endoplasmic reticulum (ER) capacity and quality control to prevent unfolded proinsulin accumulation, a major protein synthetized within the **β** cell. These signalling pathways are collectively termed unfolded protein response (UPR). The UPR machinery is required to preserve ER homeostasis and **β** cell integrity. Moreover, UPR actors play a key role by regulating ER folding capacity, increasing the degradation of misfolded proteins, and limiting the mRNA translation rate. Recent genetic and biochemical studies on mouse models and human UPR sensor mutations demonstrate a clear requirement of the UPR machinery to prevent **β** cell failure and increase **β** cell mass and adaptation throughout the progression of diabetes. In this review we will highlight the specific role of UPR actors in **β** cell compensation and failure during diabetes.

## 1. Introduction

Type 2 diabetes (T2D) mellitus is a chronic metabolic disease with “epidemic” proportions. Its global prevalence was estimated to be 6.4% worldwide (285 million adults in 2010) and is predicted to rise to approximately 7.7% (439 million) by 2030 [1]. T2D is a multifactorial disorder resulting from an interaction between genetic and environmental conditions (sedentary lifestyle and Western diet) and characterized by a peripheral insulin resistance, hyperglycaemia, and pancreatic *β* cell dysfunctions. Two defects have been reported during diabetes development, a gradual deterioration of *β* cell functions and a reduction in pancreatic *β* cells mass. *β* cell failure is not limited to T2D but is rather a common feature of all forms of diabetes, including the autoimmune type 1 diabetes (T1D), autosomal dominant onset diabetes of young (MODY), Wolfram syndrome, and Wolcott-Rallison syndrome (WRS).

In the early stage of diabetes development, the response of pancreatic islets challenged by nutrients and/or insulin resistance is a hypersecretion of insulin to maintain normoglycaemia. To this end, an adaptative and compensatory response of *β* cells is required. The process of *β* cell compensation is a combination of *β* cell mass expansion and an increase of acute glucose-stimulated insulin secretion. Postmortem analyses of pancreas of nondiabetic obese patients show an increase of *β* cell volume, implying postnatal plasticity of *β* cell mass. Moreover the *β* cell compensation process is associated with an improved capacity of the secretory machinery to support increased insulin production. Subsequently, the production of large amounts of insulin by compensating islet *β* cells places a continuous demand on the ER for proper protein synthesis, folding, trafficking, and secretion. When the folding capacity of the ER is exceeded, misfolded or unfolded proteins accumulate in the ER lumen, resulting in ER stress.

The cytoprotective response to ER stress is the unfolded protein response (UPR). Paradoxically, UPR signalling activation leads to opposite cell fates, that is, adaptation/survival versus death. Increasing evidence links the endoplasmic reticulum (ER) stress to *β* cell deterioration and apoptosis [2, 3]. Recent experiments performed in db/db mice and ob/ob mice models at different times of disease progression revealed that the maintenance (or suppression) of adaptive UPR is associated with *β* cell compensation (or failure) in obese mice [4]. Moreover, Engin et al. recently showed a progressive loss of UPR mediator expression before the onset of diabetes in NOD mice [5]. The administration of the chemical chaperone tauroursodeoxycholic acid to rescue the deleterious ER stress response improved pathophysiological signs of diabetes with a recovery of *β* cell survival and adaptation to stress [5]. In addition, the authors showed a decline of the UPR mediator in both experimental models and T2D human islets, suggesting that decreased expression of *β* cell UPR actors can play a central role in *β* cell compensation and subsequently T2D occurrence [6].

## 2. The UPR Pathway

**Figure 1:**
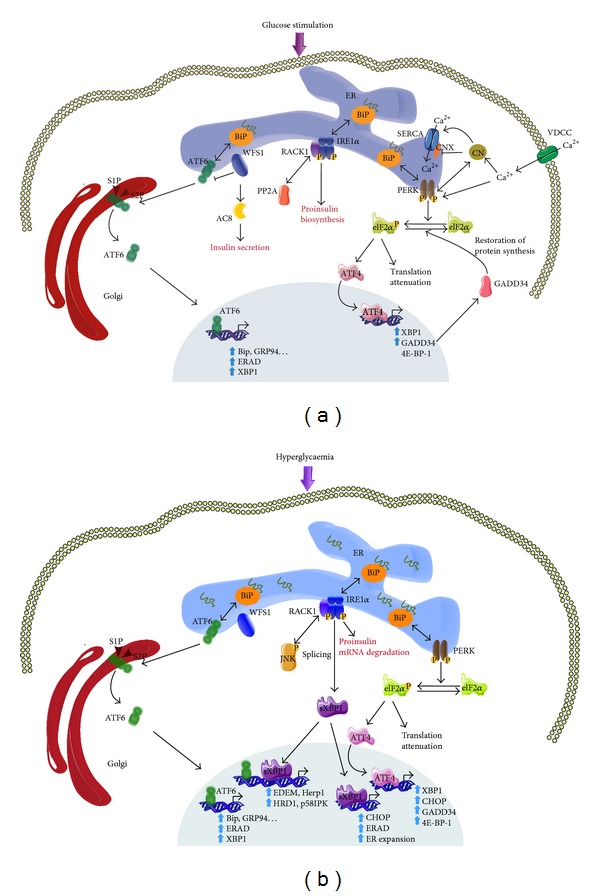
Physiological and physiopathological UPR activated pathways in *β* cells. (a) Under physiological conditions, increased proinsulin synthesis in response to postprandial glucose activates UPR to reduce ER stress and to promote *β* cell adaptation. The UPR triggers transcription of folding protein (BiP, GRP94,…), protein quality control (ERAD), UPR retrocontrol protein (GADD34), and attenuates protein translation (elF2*α*). Additionally, the UPR regulates calcium homeostasis via PERK, promotes proinsulin synthesis via IRE1*α*, and increases insulin secretion via a WFS1-AC8 pathway. (b) Under physiopathological conditions, the UPR is hyperactivated leading to IRE1*α* hyperphosphorylation, which in turn induces proinsulin mRNA degradation, JNK pathway activation, and XBP1 mRNA splicing. XBP1s alone or in synergy with ATF6 lead to expression of ER chaperon (Herp1, EDEM, HRD1, p58IPK, and ERAD) and subsequent ER expansion. Both ATF4 and sXBP1 increase CHOP mRNA expression. Under these conditions the UPR feedback is deregulated.

Three canonical ER resident molecules mediate UPR response, namely, protein kinase R-like ER kinase (PERK), inositol-requiring enzyme 1 (IRE1), and activating transcription factor 6 (ATF6), which are maintained inactive by their association with the immunoglobulin heavy chain-binding protein (BiP, GRP78) in normal conditions (Figure 1(a)). The accumulation of unfolded proteins in the ER leads to the release of PERK, IRE, and ATF6 and their subsequent activation [7, 8]. The downstream signalling effectors from these pathways converge to the nucleus and activate UPR target genes, finally reducing the ER input (Figure 1(a)). Their action is bipartite, with an acute programme that attenuates the ER workload and a latent transcriptional one that builds ER capacity.

PERK is a type 1 ER transmembrane kinase with a stress sensing luminal N-terminal domain. During ER stress PERK phosphorylates the *α*-subunit of eIF2-*α* on serine 51 leading to a delivery inhibition of the initiator methionyl-tRNAi to the ribosome and ultimately resulting in global protein translation attenuation [9] (Figure 1(a)). This phosphorylation event directly contributes to the reduction of ER stress and protects cells from ER stress-mediated apoptosis [10]. Intriguingly, the mRNA transcription of UPR target genes is selectively activated by eIF2*α* phosphorylation, as these polycistronic mRNAs have inhibitory upstream open reading frames (uPRFs) and are thus preferentially translated by the ribosome. These include the bZiP transcription factor 4 (ATF4) that acts as a regulator of UPR target genes such as C/EBP-homologous protein (CHOP) and growth arrest and DNA damage inducible gene 34 (GADD34), as well as genes involved in the redox balance and amino acid synthesis [11]. GADD34 interacts with the catalytic subunit of protein phosphatase (PP1c) and controls the level of eIF2-*α* phosphorylation by a negative feedback loop [12], allowing the restoration of an UPR basal state once ER stress is resolved.

IRE1 is a central regulator of UPR. Like PERK, IRE1 is also a type 1 transmembrane kinase with an N-terminal luminal domain that senses ER stress signalling. Two homologues of IRE1 have been described, IRE1*α* and IRE1*β*. IRE1*α* is expressed ubiquitously, showing high expression levels in the pancreas and placenta [13], whereas IRE1*β* is only expressed in the intestinal epithelium and lung [14]. IRE1 possesses kinase as well as endoribonuclease activities. Once the ER stress is triggered, IRE1 activates its RNase domain through its dimerization and transautophosphorylation and causes an unconventional splicing by the removal of 26-nucleotide intron from the X-box binding protein 1 (XBP1) mRNA (Figure 1(a)). The subsequent spliced XBP1 (XBP1s) mRNA encodes a leucine zipper transcription factor with a high transcriptional activity that upregulates genes encoding ER protein chaperones, ER associated protein degradation (ERAD), and lipid biosynthetic enzymes [15, 16]. IRE1 has also a nonspecific RNase activity that degrades mRNAs localized near the ER membrane, thereby reducing protein import into the ER lumen [17]. High levels of ER stress also activate the kinase activity of IRE1 and initiate a signalling cascade of apoptosis signal-regulating kinase 1 (ASK1)/cJun amino terminal kinase (JNK), which can participate in the apoptotic cell fate [18].

ATF6 is an ER located type 2 transmembrane protein with a basic leucine zipper DNA binding domain (Figure 1(a)). Two ubiquitously expressed isoforms of ATF6 have been described, ATF6*α* and ATF6*β* [19]. Under ER stress conditions, ATF6*α* translocates from the ER to the Golgi apparatus, where it is cleaved by Site-1 protease and Site-2 protease (S1P/S2P). The newly generated cytosolic fragment migrates to the nucleus and activates UPR gene transcription [20, 21]. The exclusive or the combined action of cleaved ATF6*α* and XBP1s is able to activate all three ER stress response elements: ERSE, UPRE, and ERSE2 [22]. ATF6*β* was first described as a repressor of ATF6*α* [23]. However, mouse embryonic fibroblasts generated from ATF6*β* null mice did not show altered UPR gene induction, suggesting a minor role for ATF6*β* in ER stress response [24]. In contrast, ATF6*α* null mice have a significant alteration in their UPR gene expression profile, suggesting a central role for ATF6*α* in ER protein quality control and protection against ER stress [24]. As the double ATF6*α*/*β* knockdown is lethal, the authors suggested that ATF6*α* and ATF6*β* provide a complementary function during early development [24]. Moreover ATF6 activity is regulated by the Wolfram syndrome 1 (WFS1) protein, which targets ATF6 to the E3 ubiquitin ligase HRD1, consequently resulting in its ubiquitination and proteasomal degradation [25]. A number of other ER stress transducers that share a high sequence homology with ATF6 have been identified, such as Luman, OASIS, BBF2H7, CREBH, and CREB4 (reviewed in [26]). However, despite their structural similarities, each ATF6 homolog seems to have specific functions in UPR regulated processes in specific organs and tissues [26].

### 2.1. *β* Cells Compensation and UPR Actors

*β* cell is a highly specialized secretory cell which responds to elevated postprandial glycaemia by increasing mRNA proinsulin translation and insulin secretion [27]. The periodic waves of proinsulin mRNA translation generate biosynthetic loads that induce UPR. To manage this burden imposed by proinsulin synthesis, *β* cells increase their ER size, as exemplified during diabetes development. In the prediabetic state, *β* cell must adapt its ER machinery to the new hyperglycaemic environment, promoting *β* cell compensation. The use of genetically modified mouse models and genetic studies from human diabetic patients demonstrated that UPR actors support this adaptation as well as *β* cell compensatory mechanism [4, 28].

### 2.2. Insulin Mutants and *β* Cells Compensation

Misfolding of proinsulin is associated with ER stress and severe dysfunctions leading to a massive destruction of *β* cells.* In vivo* evidences were observed in both Akita and Munich mice carrying cysteine residues mutations that interfere with disulphide bond formation [29, 30]. Interestingly, inactivation of the UPR induced proapoptotic Chop gene delayed the onset of diabetes in heterozygous Akita mice, suggesting a key role for CHOP in ER stress-mediated *β* cell apoptosis [30]. Diabetes of youth (MIDY) is a syndrome caused by a heterozygous mutation of the coding sequence of proinsulin leading to an autosomal-dominant and insulin-deficient diabetes [31]. This mutation has been shown to be the second most common cause of permanent neonatal diabetes related to ER stress [32, 33]. In line with these observations, inducible expression of the human analogue proinsulin C96Y mutation of Akita mice in rat insulinoma-1 (INS-1) caused ER stress and cell apoptosis. However, upregulation of UPR and ERAD seems to have a protective effect [34].* In vivo* expression of the same proinsulin mutant driven by the weak Ins1 promoter induced both ER stress and pancreatic compensation [35]. Altogether these data demonstrate a clear link between misfolding of proinsulin and ER stress induction.

### 2.3. PERK-elF2*α*-ATF4 Pathway in *β* Cells Compensation

Loss of function mutation in the EIF2AK3 gene-encoding PERK was associated with WRS, which has been confirmed by the functional characterisation of* Perk* knockout mice [36]. Embryonic development of these mice is normal but they exhibit a postnatal growth retardation, skeletal dysplasia, and progressive loss of *β* cells, associated with defects in ER secretory machinery and proinsulin folding [37, 38]. However, generation of *β* cell specific Perk knockout mice revealed that *β* cell death was not increased, but rather *β* cell proliferation and differentiation were repressed during the embryonic and postnatal state [39]. Other studies on these mice models demonstrated an impaired ER to Golgi anterograde trafficking, retrotranslocation out of the ER, and proteasomal degradation, showing requirement of PERK for ER and Golgi integrity and processing of ATF6 [40]. In contrast to the first rapport, specific *β* cell inducible* Perk* deletion in mice showed a rapid progression of insulin dependent diabetes regardless of mice age [41]. The authors showed, on the one hand, that this phenotype was due to an increased *β* cell proliferation after the induction of PERK deletion due to the increased activation cyclin D-dependent kinase activity. On the other hand, they showed a significant increase in *β* cell death, associated with an activation of other UPR actors and a disturbance in calcium homeostasis [41]. Moreover, a recent work demonstrated that PERK, in concert with calcineurin, regulates ER calcium reuptake through calnexin interaction and a negative regulation of the sarcoplasmic endoplasmic reticulum calcium ATPase pump (SERCA) [42] (Figure 1(a)). PERK thus appears to sense glucose by direct sensing of ER calcium levels, raising the possibility that the primary function of PERK in *β* cell is to modulate proinsulin quality control and trafficking.

Within the *β* cell, phosphorylation of elF2*α* is mostly PERK dependent in ER stress conditions (Figure 1(b)). Mice harbouring a homozygote knock-in mutation at the PERK-mediated phosphorylation site (Ser51Ala) of elF2*α* display a severe *β* cell deficiency detectable in late stage embryos and die within 18 h after birth as a consequence of hyperglycaemia associated with defective neoglucogenesis [43]. Heterozygote mice development is normal; however, when challenged with a high fat diet, they develop severe obesity, glucose intolerance, and impaired insulin release. In this genetic context reduced insulin content, fewer granules, ER distension, and a prolonged association of proinsulin with BiP have been observed. Thus, elF2*α* phosphorylation is required for UPR to prevent *β* cell failure when insulin demand is increased. These findings demonstrate a central role of elF2*α* phosphorylation in *β* cell adaptation during compensation [44]. The generation of conditional homozygote ser51Ala elF2*α* mutation in *β* cell confirmed this observation. These mice exhibit a high rate of *β* cell apoptosis, likely caused by hypoinsulinemia, severe glucose intolerance, and hyperglycaemia. Furthermore, the authors showed ER distension and mitochondrial damage associated with a lower basal expression of the majority of UPR genes and *β* cell antioxidant responsive genes. Altogether these data indicate that the correct UPR and antioxidant response controlled by PERK-elF2*α* signalling are required for *β* cell adaptation and survival [45].

Phosphorylation of elF2*α* leads to attenuate global translation of most mRNA although translation of ATF4 is selectively stimulated in this context [11] (Figure 1(b)). The role of ATF4 in insulin secretion and *β* cell survival is controversial. Pioneer studies showed that* Atf4* knockout mice on a 129SV genetic background are lean, hypoglycaemic, and resistant to diet-induced obesity, probably as a result of increased energy expenditure. However, no effect was observed on plasma insulin level when mice were fed with normal diet, whereas insulin levels were shown to be lower in* Atf4* null mice compared to wild-type mice when fed with a high fat diet [46, 47]. Paradoxically, other studies using* Atf4* KO mice on C57BL6/J genetic background demonstrated that these mice are hypoglycaemic and hyperinsulinemic with an increased *β* cell mass and function. The phenotype of these mice could be secondary to an overexpression of osteocalcin in osteoblasts, a bone derived secreted molecule promoting insulin secretion and insulin sensitivity [48]. The genetic background may explain the differences observed in these Atf4 null mice models. The role of ATF4 in insulin synthesis and *β* cell adaptation to ER stress remains unclear, and further studies using a *β* cell specific* Atf4* KO mouse model may be useful to answer this question.

ATF4 activation by elF2*α* also leads to the transcription of elF4-E binding protein (4E-BP-1), a well-documented gene involved in *β* cells adaptation to stress [49]. In fact, translation attenuation by elF2*α* phosphorylation is transient, subsequently leading to the feedback dephosphorylation by GADD34, whereas 4E-BP1 suppresses prolonged translation by the inhibition of cap-dependent translation [49, 50]. *β* cell specific 4e-bp1 KO mice are normal without any metabolic disorder when fed normal diet but are insulin resistant and show *β* cell defects under high fat diet due to induced ER stress [49, 51, 52]. Moreover, the inactivation of 4e-bp1 gene in Min6 cell line results in sensitization to ER stress and increased *β* cell loss and hyperglycaemia in diabetic mouse models [49]. These findings suggest a central role of 4E-BP1 in *β* cell adaptation to ER stress. In contrast, other groups indicated that suppression of 4E-BP1 expression is involved in beneficial effects of high-density lipoproteins on *β* cells survival [53], suggesting that the role of ATF4 in *β* cell compensation might depend on several cellular interactions.

### 2.4. IRE1*α*-XBP1 Pathways in *β* Cells Compensation

IRE1*α* is the major isoform expressed in the pancreas and plays a central role in *β* cell adaptation to ER stress (Figure 1(b)). IRE1*α* is required for embryonic development as demonstrated by the embryonic lethality of global IRE1*α* KO mice. IRE1*α* plays a crucial role in insulin biosynthesis. The generation of IRE1*α* conditional KO mice revealed that IRE1*α* deletion caused mild hyperinsulinemia and hyperglycaemia and a lower body mass under normal diet [54]. Physiological activation of UPR by glucose results in IRE1*α* phosphorylation, without increasing XBP1 mRNA splicing. Moreover, knockdown of IRE1*α* in INS-1 insulinoma cell line resulted in decreased proinsulin biosynthesis or insulin content without impacting global protein synthesis or insulin secretion, suggesting a beneficial effect of IRE1*α* activation by transit exposure to glucose in *β* cells [55]. However, chronic exposure to high glucose leads to hyperphosphorylation of IRE1*α*, which in turn results in selective degradation of proinsulin mRNA [56]. This may be part of the *β* cell protective mechanism from apoptosis under chronic hyperglycaemia induced ER stress. This adaptative mechanism combined to UPR activation may explain the reduced insulin secretion in type 2 diabetic patients in the absence of *β* cell death. IRE1*α* dephosphorylation is mediated by proteins phosphatase A2 (PP2A) through ternary complex containing the scaffold protein RACK1 (receptor for activated C kinase 1). Under glucose stimulation or ER stress, RACK1 mediates IRE1*α*, RACK1, and PP2A complex formation and promotes IRE1*α* dephosphorylation by PP2A, thereby inhibiting IRE1*α* activation and attenuating IRE1*α*-dependent increase in insulin production. Moreover, IRE1*α* activation is increased and RACK1 abundance is decreased in db/db mice [57]. The endoplasmic activity of IRE1*α* is also involved in the activation of a key metabolic enzyme, AMP-activated kinase (AMPK), in response to nitric oxide (NO) and ER stress in *β* cells [58]. AMPK is a holoenzyme activated by changes in AMP/ATP ratio, shifting from glucose to the use of lipids as an energy source in order to respond to cellular demand [59]. Activated AMPK by GTPase dynamin related protein 1 (DRP1) phosphorylation prevents ER and mitochondrial alteration in stressed *β* cells [60]. In addition IRE1*α* modulates nuclear factor *κ* light chain enhancer (NF-*κ*B) target gene expression and IL-1*β* activation under mild ER stress, which could contribute to chemokine-induced *β* cell death [61].

Upon UPR mediating IRE1*α* activation, XBP1 splicing is the major event. Several reports indicated that XPB1s target genes and its downstream effect are cell specific and might be dependent on the activating pathways. Like IRE1*α*, XBP1 deficient mice died between 10.5 and 14.5 day after birth because of cardiac myocyte defects [62]. Heterozygous Xbp1 mice exhibited significant increase in body mass associated with a progressive hyperinsulinemia and glucose intolerance when fed with a high fat diet [63]. These mice showed increased ER stress and decreased insulin receptor expression in the liver. The *β* cell-specific deletion of XBP1 in mice resulted in a modest hyperglycaemia and glucose intolerance caused by decreased insulin secretion [64]. The loss of XBP1 markedly decreased the number of insulin granules and impaired proinsulin processing. Further analysis revealed that XBP1 deficiency not only participated in the ER stress in *β* cells but also caused constitutive hyperactivation of its upstream activator, IRE1*α*, which could degrade a subset of mRNAs encoding proinsulin-processing enzymes [64]. In summary, *β* cell defects in XBP1 mutant mice result from a combined effect of XBP1 suppression on canonical UPR and its negative feedback activation of IRE1*α*. Altogether these findings suggest a dual and opposite role for IRE1*α* in *β* cells. A precise regulated feedback circuit involving IRE1*α* and its product XBP1s is required to achieve optimal insulin secretion and glucose control. In contrast, sustain production of XBP1s leads to inhibition of PDX1 and MAFA expressions, promoting *β* cells dysfunction and apoptosis [54].

### 2.5. ATF6 Pathways in *β* Cell Compensation

Both ATF6 isoforms are required for positive regulation of UPR. However, the transcriptional activity of ATF6*β* is lower than that of ATF6*α*. The double knockdown of the two isoforms caused an embryonic lethality demonstrating overlapping functions of ATF6*α* and ATF6*β*, which are essential for embryonic development [24]. ATF6*α* KO mice demonstrated a severe hypoglycaemia suggesting that suppression of ATF6*α* increased insulin sensitivity [65]. Treatment of these mice with a pharmacological ER stress inducer leads to liver dysfunction and steatosis [66]. Furthermore, when fed with a high fat diet, ATF6*α* null mice developed insulin resistance associated with impaired insulin secretion and lower insulin content, reinforcing the idea of a key role of ATF6*α* in *β* cells adaptation and insulin resistance [65]. Recently, a basal expression of active ATF6*α* was demonstrated to be essential for *β* cell survival even under unstressed conditions. Interestingly, specific functions of ATF6*α* have been revealed depending on its interaction with XBP1. When ATF6*α* is acting alone, it induces the expression of a cluster of genes involved in protein folding such as BiP and GRP94. When it heterodimerizes with XBP-1 they are modulating the expression of specific class of target genes, such as genes involved in protein degradation (EDEM, Herpude1, HRD1, and p58IPK) [24]. In contrast, a deleterious effect of active ATF6*α* overexpression on *β* cell function and expression of insulin, PDX1, and MAFA in INS-1 cells was shown [67]. Interestingly, some reports demonstrated that some ATF6 variants are associated with type 2 diabetes and new onset diabetes after transplantation (NODAT), suggesting potential links between ATF6*α* and human diabetes pathophysiology [68, 69]. It is important to note that, from the myriad of ATF6 homolog described until now, only old astrocyte specifically induced substance (OASIS) was identified in *β* cell [70]. However, microarray analysis of INS-1 *β* cell line overexpressing the active form of OASIS showed its implication in extracellular matrix production and protein transport but not in the classical ER stress response [70].

A great interest has been focused on the ATF6*α* negative regulator WFS1 because of its association with the Wolfram syndrome, a rare genetic disorder [71, 72]. Loss of function mutation in the* wfs1* gene encoding wolframin protein caused neurodegenerative disorders characterised by juvenile onset diabetes mellitus, optic atrophy, and hearing impairment [73, 74]. WFS1 KO mice exhibit an activated ER stress especially in *β* cells, leading to *β* cell loss through impaired cell cycle progression and increased apoptosis [75]. Conditional WFS1 knockdown in *β* cell induced diabetes as a result of enhanced ER stress and apoptosis [76]. Moreover WFS1 is essential for glucose and glucagon-like peptide 1 (GLP1) stimulated AMP production and regulation of insulin biosynthesis and secretion [77]. Under glucose stimulation, WFS1 translocates from the ER to plasma membrane, where it stimulates cyclic adenosine monophosphate [73] synthesis through an interaction with adenylyl cyclase 8 (AC8), which subsequently promoted insulin secretion [77] (Figure 1(a)). A recent report using induced pluripotent stem (iPS) cells to create *β* cells from individuals with Wolfram syndrome confirmed these observations. In this study, WFS1 deficient *β* cells showed increased levels of UPR genes and decreased insulin content, leading to *β* cell dysfunctions as previously described in mouse models [78].

### 2.6. The UPR/ER Stress Induction of *β* Cell Apoptosis

As discussed above, the UPR regulates both survival and death effectors. It is now clear that the three unfolded protein sensors—IRE1a, PERK, and ATF6—influence the life-death decision. The inability of UPR outputs to restore homeostasis may generate continuous signalling from these sensors, tipping the balance in favour of apoptosis. The ER might actually serve as a site where apoptotic signals are generated and integrated to elicit the death response. ER stress leads to apoptosis by activating both mitochondrial dependent and independent pathways. Several stimuli have been linked to ER stress-induced apoptosis including hyperglycaemia, exposure to long-chain free fatty acids (e.g., palmitate) [79–81], hyperinsulinemia occurring in the prediabetic stage [82], glucose deprivation [83], islet amyloid polypeptide (IAPP) expression [84], and exposure to inflammatory cytokine [85]. Players involved in the cell death response include PERK/elF2*α*-dependent transcriptional induction of proapoptotic transcription factor CHOP which represses Bcl-2 [86], IRE1-mediated activation of ASK1/JNK [18], and cleavage and activation of procaspase 12 (caspase 4 in humans) [87, 88].

CHOP has retained a special attention as a central mediator of apoptosis. Its expression is low under physiological condition but is strongly induced upon ER stress [89]. The induction of CHOP is regulated by ATF4 [11, 90] and ATF6 [91–93] and its role in ER stress-induced apoptosis was demonstrated both* in vitro* and* in vivo* [94]. Mice lacking CHOP are protected from renal toxicity of the ER stressor tunicamycin, an inhibitor of glycosylation [94]. CHOP deletion promotes *β* cells survival in both genetic and diet-induced insulin resistant mice models [30, 92]. Pancreatic *β* cells are also sensitive to oxidative stress, but *β* cells from CHOP knockout mice are protected and maintain insulin secretion under oxidative stress [92, 95]. Moreover, islets from these mice showed resistance to NO, a chemical agent implicated in *β* cells disruption in type 1 diabetes [96]. In contrast, CHOP deficiency in a genetic background of nonobese diabetic mice (NOD-Chop−/−) did not affect the development of insulitis, diabetes, and *β* cells apoptosis [97]. Interestingly, CHOP knockout mice on a C57BL/6 background showed a different phenotype, with abdominal obesity and hepatic steatosis, while preserving normal glucose tolerance and insulin sensitivity [98].

Under ER stress CHOP positively regulates the expression of genes involved in apoptosis including GADD34 [50, 99], the ER oxidoreductin 1 *α* (ERO1*α*) [100], death receptor 5 (DR5) [101], and the pseudokinase tribbles related 3 (TRB3) [102]. As shown for CHOP deletion, genetic inactivation of these genes protected against *β* cell ER stress-induced apoptosis [100, 103–105]. Additionally, CHOP represses the expression of the antiapoptotic gene Bcl2 and enhances oxidant injury [106]. Finally, deletion of CHOP was reported to prevent the cytokine-mediated cleavage of caspase 9 and caspase 3 and subsequent *β* cells apoptosis by reducing cytokine-induced NF-*κ*B activity and the expression of key NF-*κ*B target genes involved in apoptosis and inflammation [107].

ER stress-mediated apoptosis can also be signalled through IRE1*α* dependent activation of JNK pathway [18]. IRE1*α* interacts with TBF receptor associated factor 2 (TRAF2) and ASK1 mediating JNK phosphorylation [18, 108]. The analysis of ASK1 deficient mice showed that ASK1 loss of function attenuated insulin resistance, cardiac inflammation and fibrosis, vascular endothelial dysfunction, and remodelling induced by diet-induced obesity [109]. Moreover, deletion of ASK1 in homozygous Akita mice protected *β* cells from ER induced apoptosis and delayed the onset of diabetes [110]. The IRE1*α*/TRAF2 complexes also contributes to ER stress-induced apoptosis by promoting the clustering of procaspase-12 and its activation by cleavage in response to ER stress [111]. In addition, the IRE1*α*/TRAF2 complex interacts with IKK, an inhibitor of NF-*κ*B, mediating its activation and promoting cell apoptosis in response to ER stress [112, 113]. Finally, members of Bcl2 family including BAX, BAK, BIM, and PUMA have been reported to directly interact with IRE1*α* demonstrating a physical link between members of the core apoptotic pathway and the UPR [114, 115]. In contrast, IRE1*α* forms a stable protein complex with Bax inhibitor-1 (BI-1) protein, suppressing cell death [116]. The IRE1*α*/BI-1 association decreased the ribonuclease activity of IRE1*α* and seemed to be required for early adaptive responses against ER stress-induced apoptosis [116]. The control of IRE1*α* activity appears to be central in the mechanism protecting *β* cells from ER stress-induced apoptosis. Further studies are needed to understand the various aspect of IRE1*α* regulation and the contribution of the others actors of UPR in the ER stress-induced apoptosis.

### 2.7. The UPR in Human Diabetes

Clear evidence of the existence of ER stress in human *β* cells has been reported in the last decade [2, 3, 6, 117]. First analysis of islets from human T2D patients showed an ER extension but modest signs of ER stress marker in human pancreatic samples and isolated islets. However, glucose stimulation induced increased UPR in T2D islets cells [3]. Some markers of ER stress are increased in T1D human islets with partial ER stress [117, 118]. A recent report shows an alteration in the expression of specific branches of UPR mediators in T2D *β* cells [6]. These findings support the hypothesis of a decline in *β* cell adaptation/compensation during the progression of diabetes in human.

## 3. Conclusion

The *β* cell has a marked capacity to adapt to environment changes by increasing its mass and function. Diabetic signs occur when this adaptative mechanism fails to compensate for the increasing insulin demand. Activation of UPR actors is triggered in the early stage of the compensatory mechanism and may play a central role in *β* cell adaptation and subsequent functions. Further studies are required to understand the physiological significance and the direct implication of ER stress and UPR in the early stage of diabetes physiopathology. Moreover, the relationships among UPR actors, their activation, and *β* cell fate (adaptation/survival versus *β* cell dysfunction/apoptosis) remain to be fully clarified. Theoretically, the size of *β* cell mass is controlled by a balance between proliferation and apoptosis. Either increase of *β* cell apoptosis or decrease in *β* cell adaptation and compensation could, therefore, reduce the *β* cell mass in T2D patients. Studies carried out during diabetes development are required to better understand the mechanism of compensatory capacity and subsequent *β* cell loss in humans. This is of particular interest, since it could have beneficial impact for the treatment of metabolic diseases such as diabetes.

It is important to note that most UPR molecules have an adaptive function in *β* cells. Their role in the switch from survival to apoptosis is clearly demonstrated* in vitro* and in animal models but it remains unclear whether the same mechanisms occur in human *β* cell. Isolating and culturing primary *β* cells may be very stressful and do not perfectly reflect the* in vivo* context. Therefore the use of alternative method such as immunohistochemistry is powerful to determine the role of each branch of UPR in diabetes.
